# Contribution of sublinear and supralinear dendritic integration to neuronal computations

**DOI:** 10.3389/fncel.2015.00067

**Published:** 2015-03-24

**Authors:** Alexandra Tran-Van-Minh, Romain D. Cazé, Therése Abrahamsson, Laurence Cathala, Boris S. Gutkin, David A. DiGregorio

**Affiliations:** ^1^Unit of Dynamic Neuronal Imaging, Department of Neuroscience, CNRS UMR 3571, Institut PasteurParis, France; ^2^Group for Neural Theory, LNC INSERM U960, Institut d’Etude de la Cognition de l’Ecole normale supérieure, Ecole normale supérieureParis, France; ^3^Department of Bioengineering, Imperial College LondonLondon, UK; ^4^Center for Research in Neuroscience, Department of Neurology and Neurosurgery, The Research Institute of the McGill University Health Centre, Montreal General HospitalMontreal, QC, Canada; ^5^Sorbonne Universités, UPMC Univ Paris 6, UMR 8256 B2A, Team Brain Development, Repair and AgingParis, France; ^6^Federal Research University Higher School of EconomicsMoscow, Russia

**Keywords:** dendrites, neural computation, nonlinear transformations, Boolean analysis, binary neruons, uncaging, input-output transformation, votlage activated channels

## Abstract

Nonlinear dendritic integration is thought to increase the computational ability of neurons. Most studies focus on how supralinear summation of excitatory synaptic responses arising from clustered inputs within single dendrites result in the enhancement of neuronal firing, enabling simple computations such as feature detection. Recent reports have shown that sublinear summation is also a prominent dendritic operation, extending the range of subthreshold input-output (sI/O) transformations conferred by dendrites. Like supralinear operations, sublinear dendritic operations also increase the repertoire of neuronal computations, but feature extraction requires different synaptic connectivity strategies for each of these operations. In this article we will review the experimental and theoretical findings describing the biophysical determinants of the three primary classes of dendritic operations: linear, sublinear, and supralinear. We then review a Boolean algebra-based analysis of simplified neuron models, which provides insight into how dendritic operations influence neuronal computations. We highlight how neuronal computations are critically dependent on the interplay of dendritic properties (morphology and voltage-gated channel expression), spiking threshold and distribution of synaptic inputs carrying particular sensory features. Finally, we describe how global (scattered) and local (clustered) integration strategies permit the implementation of similar classes of computations, one example being the object feature binding problem.

## Introduction

In order to control behavior, the brain relies on the ability of its neuronal networks to process information arising from external and internal sources. How single neurons decode combinations of sensory features and transform them into a spiking output is still unknown, and represents a subject of intense study. The complexity of the single neuronal coding problem can be illustrated by the paradoxical finding that neurons exhibiting narrowly tuned receptive fields often appear to be driven by synaptic inputs that themselves are broadly tuned (Chadderton et al., 2014). One hypothesis is that nonlinear dendritic transformations are critical for such neuronal computations. Decades of experimental and modeling studies on dendrites have led to the consensus that active properties of dendrites are primarily responsible for nonlinear integration, in particular **supralinear** operations (Mel, 1994; Spruston and Kath, 2004; Johnston and Narayanan, 2008). Nonetheless other findings indicate that **sublinear** integration of synaptic inputs is possible in multiple neuron types, and results from either active (Cash and Yuste, 1998; Hu et al., 2010) or passive dendritic properties (Abrahamsson et al., 2012; Vervaeke et al., 2012).

What is the evidence that nonlinear dendritic properties contribute to neuronal computations? Numerical simulations suggest that supralinear dendritic operations are essential for translation-invariant orientation tuning (Mel et al., 1998) and binocular disparity tuning (Archie and Mel, 2000), while sublinear dendritic operations contribute to coincidence detection of auditory stimuli (Agmon-Snir et al., 1998). Recently, state-of-the-art *in vivo* recordings have shown that dendritic supralinearities are associated with various other neuronal computations: formation of hippocampal place fields (Lee et al., 2012), detection of multi-modal sensory stimuli (Xu et al., 2012), angular tuning of barrel cortex pyramidal neurons (Lavzin et al., 2012), and enhancement of orientation tuning (Smith et al., 2013). Sublinear operations have also been shown to underlie orientation selectivity of binocular neurons in visual cortex *in vivo* (Longordo et al., 2013).

Nevertheless, a direct link between the dendritic transformations and the associated neuronal computations is still lacking. Analytical methods implementing mathematical approximations of measured dendritic operations can be used to make estimates of the possible number and type of neuronal computations. For example, binary neuron models were used to quantify what was previously shown with biophysical models (Mel, 1994), namely that nonlinear dendrites support a larger number of neuronal computations (Poirazi and Mel, 2001; Cazé et al., 2013). Such simplifications can provide analytical insight and make testable predictions as to which computations are made possible by dendritic operations. Moreover, analytical methods show under which conditions the expanded computational capacities are generic, i.e., not tied to the specific example parameters of the biophysical model.

Here we review the biophysical determinants of different classes of dendritic operations (linear, sublinear and supralinear), how they are measured experimentally, and finally, using a recently published Boolean-based analysis of equivalent dendritic trees (Cazé et al., 2012, 2013, 2014), we review how these operations combine with other cellular properties to determine neuronal computations.

## Dendritic Integration

Neurons integrate synaptic inputs arriving primarily on dendritic trees carrying information from presynaptic neurons, by transforming them into synaptic potentials using a variety of cell-specific synaptic and cellular mechanisms. During synaptic transmission, the activation of neurotransmitter-gated conductances results in either a transient depolarization or hyperpolarization of the postsynaptic membrane potential. When the net depolarization resulting from **synaptic integration** of multiple synaptic inputs is greater than the spike threshold potential, the neuron generates an **action potential** (AP), or **spike**. Synaptic integration is a critical determinant of **neuronal computations**, the process by which a postsynaptic neuron transforms presynaptic information (coded in input activation patterns) into an output signal (encoded in a firing pattern) (Häusser and Mel, 2003; London and Häusser, 2005; Silver, 2010; Larkum, 2013; Chadderton et al., 2014). This review will focus primarily on the integration of excitatory post-synaptic potentials (EPSPs) mediated by ionotropic glutamate receptors.

**Figure 1:**
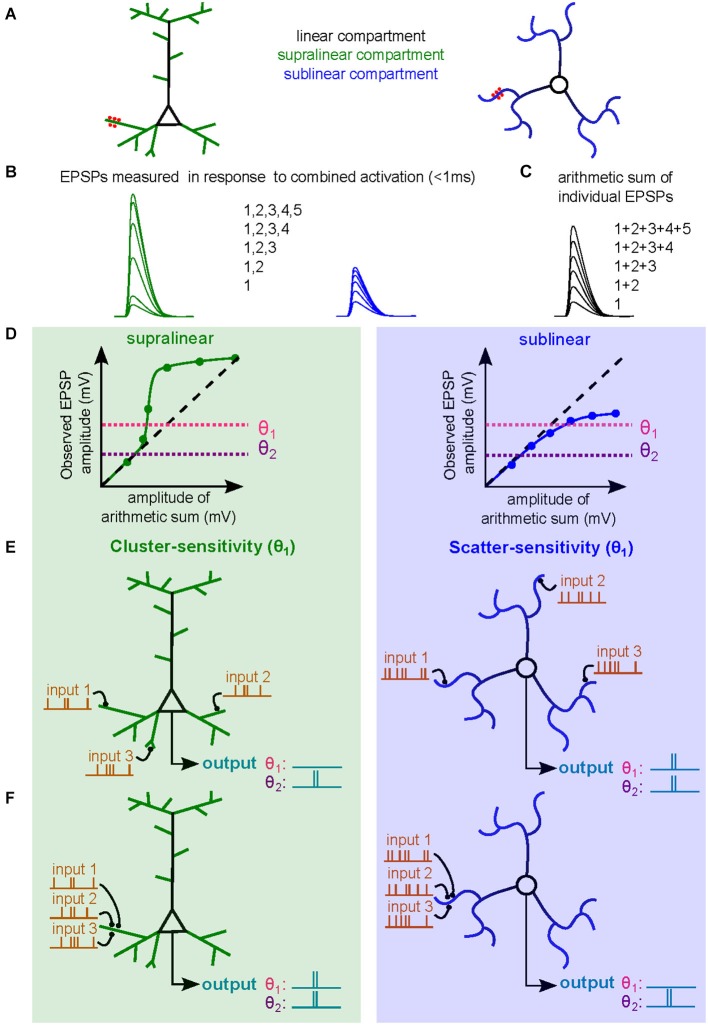
**Dendritic operations and their influence on neuronal firing. (A)** Schematic diagram of a subthreshold synaptic input-output experiment in a neuron with supralinear dendritic compartments (*left*, supralinear compartments in green, linear compartments in black) or in a neuron with sublinear dendritic compartments (*right*, sublinear compartments in blue). The red spots are sites of synaptic activation or sites of glutamate uncaging. **(B)** Somatic voltage responses evoked by simultaneous synaptic activation or uncaging. Green curves are responses evoked with increasing number of synapses activated within a supralinear dendrites. Blue traces are similarly obtained within a sublinear dendrite. **(C)** Arithmetic sum of individual responses to synaptic activation or uncaging. **(D)** Subthreshold input/output relationships (sI/O) used to quantify dendritic operations. The dashed line represents a linear releationship. Two horizontal dotted lines indicate two example somatic spike thresholds (θ_1_ and θ_2_). **(E,F)** Example of synaptic integration of three synaptic inputs distributed across the dendritic tree **(E)** or clustered on a single dendritic branch **(F)** of a neuron with supralinear dendritic compartments (*left*) or sublinear compartments (*right*). The output spike train, and hence neuronal computation, differs depending on the threshold. The more depolarized threshold value (θ_1_) allows the neuron with supralinear dendrites to exhibit a cluster-sensitive neuronal computation (fires only when three inputs are activated in the same compartment). The θ_1_ threshold also allows a neuron with sublinear dendrites to exhibit scatter-sensitive neuronal computations. The lower threshold (θ_2_) imparts a different neuronal computation based on simple linear summation and is not sensitive to activated synapse location.

Dendritic integration can be quantified by comparing the **observed** depolarization resulting from the simultaneous activation of the same synaptic inputs (Figure 1B), also called a compound EPSP, and the arithmetic sum of individual EPSPs (**expected** membrane depolarization) (Figure 1C). The dendritic subthreshold input-output (sI/O) relationship is easily described by plotting observed vs. expected depolarizations for different numbers of co-activated synapses (Figure 1). Mathematical functions can be used to describe the **operation** performed. The sI/O relationships fall into three categories of dendritic operations: (1) **linear**, where the observed depolarization equals the expected depolarization; (2) **supralinear**, where the observed depolarization exceeds the expected depolarization (above the linear line; Figure 1D, left); and (3) **sublinear**, where the observed depolarization is less than the expected depolarization (below the linear line; Figure 1D, right). Much of the experimental evidence of nonlinear integration suggests dendrites perform **supralinear** operations, resulting from the contribution of active dendritic conductances (Mel, 1994; Johnston and Narayanan, 2008; Spruston, 2008). Recent studies suggest that **sublinear** operations could be mediated solely by passive properties (Abrahamsson et al., 2012; Vervaeke et al., 2012), while other studies have shown that activation of potassium channels can produce sublinear summation (Cash and Yuste, 1999; Hu et al., 2010). The detailed biophysical mechanisms determining specific dendritic operations are discussed in depth below.

The type of **dendritic operation** strongly contributes to the nature of the resultant **neuronal computation**. For example, co-activation of synapses within a single electrical compartment that exhibits supralinear integration will produce dendritic voltage signals that are larger than expected due to amplification by activation of voltage-sensitive channels. This large depolarization is thereby more likely to drive the neuron to spike threshold. The resulting sI/O will reflect a neuronal computation that is **cluster sensitive** (Figures 1E,F, left, θ_1_). For a neuron with sublinear dendrites, clustered synaptic activity will be less efficient at triggering a spike than if the same inputs were distributed in different compartments, thus promoting computations that are **scatter sensitive** (Figures 1E,F, right, θ_1_; Cazé et al., 2013). Such neuronal computations enable the discrimination of patterns of synaptic activation with different levels of spatial and temporal correlations, which could not be otherwise performed by linear dendrites (Mel, 1992). Nevertheless, it should be noted that the dendritic operation is insufficient to define the computation, synaptic placement and spike threshold also influence the final neuronal computation. In Figures 1D–F we show that lowering the spike threshold (θ_2_) would restrict the access to only the linear regime of the subthreshold dendritic operation. Finally, ongoing synaptic activity can occur in the presence of AP firing, and thus constitutes supra-threshold synaptic integration (Silver, 2010), which we will not address in this review.

## Biophysical Mechanisms Influencing Synaptic Integration

### Effect of Passive Membrane Properties on EPSPs Summation

**Figure 2:**
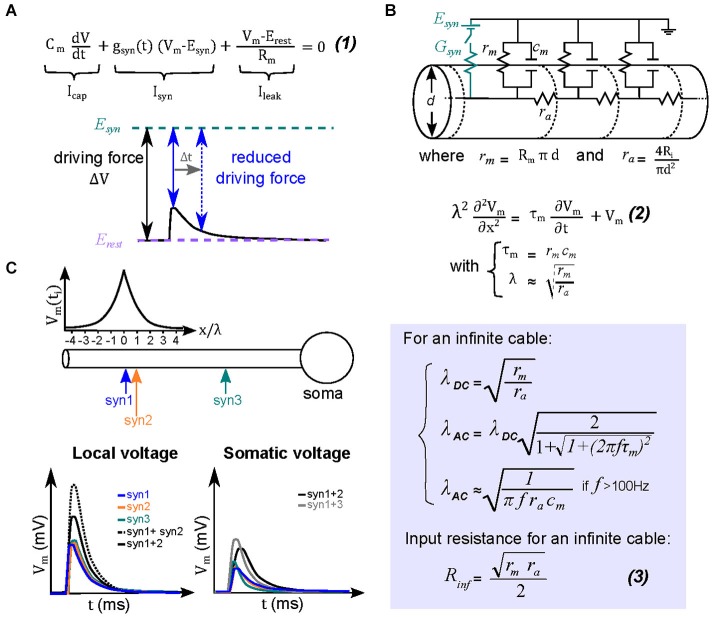
**Theoretical basis for sublinear summation within passive dendrites. (A)** Equation (1) describes the different current components underlying an EPSP in a single electrical compartment. Integration of this equation describes the variation of the membrane voltage over time. The transient change in driving force (ΔV = V_m_ − *E*_syn_) is determined by the amplitude and time course of the local dendritic EPSP (black trace). At the peak of the EPSP (solid blue arrow) the driving force is maximally reduced, and then recovers back to that at resting membrane potentials during the EPSP decay (dotted blue arrow). The reduced driving force decreases the synaptic current, and hence the net depolarization, creating a sublinear relationship between EPSP and its underlying conductance. **(B)** Equivalent circuit for dendritic cables, where g_m_ and c_m_ are the membrane conductance and capacitance, respectively, and r_a_ is the axial resistance of a unit of cable. A synapse is represented in the circuit (by the synaptic conductance G_syn_ and the synaptic reversal potential E_syn_). For an infinite cable, the spatio-temporal distribution of voltage is described by the relation (2), where τ_m_ is the membrane time constant, and λ is the length constant. The length constant relationships are derived from solving the cable equation (2) for step changes in membrane voltage (λ_DC_) or for a sinusoidal membrane potential change (λ_AC_). The latter is helpful to understand the dendritic filtering of transient EPSPs. Equation (3) is the relation for the input resistance R_D_ for an infinite cable. **(C)** *Top*, ball-and-stick model of a neuron with colored arrows indicating the location of three synapses (Syn 1–3). The graph above the diagram represents the peak amplitude of a dendritic EPSP as a function of distance. *Bottom*, the two graphs describe respectively the dendritic and somatic depolarizations in response to individual (colored lines) or combined synaptic inputs (black lines). Concomitant activation of two neighboring synaptic inputs (within ~λ_AC_) will therefore mutually reduce their driving force and sum sublinearly (for example synapses 1 and 2, solid black trace for the EPSP observed in response to their simultaneous activation, dashed black trace for the arithmetic sum of the individual EPSPs). More separated synapses will, however, sum more linearly (synapses 1 and 3, gray trace).

Because neurons communicate with each other using electrical signals, the analysis of their signaling properties is generally performed using principles of electrical circuits. A **single compartment** equivalent circuit describes well the electrical behavior of a cell without any dendrite or active properties. Four parameters determine the amplitude and time course of the EPSP: a transient synaptic conductance (G_syn_), the electromotive force of its ion flux (driving force), the membrane resistance (specific membrane resistance; R_m_), and the specific membrane capacitance (C_m_). The difference between the membrane potential and the reversal potential for G_syn_ sets the driving force (V_m_ − *E*_rev_; Figure 2A), thus as G_syn_ increases, I_syn_ increases, and V_m_ becomes more depolarized. For large conductances, V_m_ approaches E_rev_ and the driving force is reduced, resulting in decreased current flow for the same G_syn_ (Figure 2A). This results in a sublinear relationship between G_syn_ and EPSP size. Since quantal synaptic conductances are generally small, it is when multiple synapses are activated simultaneously that the driving force decreases sufficiently to produce sublinear integration (Figure 2C). Therefore, for passive single compartment model cells, synaptic summation is already essentially sublinear, which was first demonstrated at the neuromuscular junction (Martin, 1955).

More complex, but also more realistic, equivalent circuit models take into account neuronal morphology, such as dendritic arborizations. Wilfrid Rall pioneered the use of such multi-compartmental equivalent circuit models in order to study synaptic integration in neurons with **passive** dendrites. His primary advance was to consider dendrites as electrical cables (Rall, 1967) that contained an additional parameter, the axial resistance (r_a_), which electrically couples multiple elementary single compartment models (Figure 2B). Because each elementary compartment will allow current to leak across the membrane, the current injected in the next compartment (across r_a_) decreases progressively as it travels along the cable or dendrite, which results in an attenuation of the local EPSP amplitude and a slowing of its time course. Such **dendritic filtering** accounts for why local EPSPs in dendrites tend to be larger and faster than those recorded in the soma. It therefore follows that more distal synaptic inputs (for a given G_syn_) would result in a progressively smaller somatic depolarization and thus a smaller influence on the firing output of a neuron (Rinzel and Rall, 1974; Magee and Cook, 2000; Spruston, 2008). Also in dendrites the** local input resistance (R_D_) or impedance** (**Z_D_**; to account for the effect of capacitance on fast time-varying inputs) increases with increasing distance from the soma due to a diminished shunt effect of the soma and the high resistance of the sealed cable (Rinzel and Rall, 1974). We will henceforth refer to Z_D_, since it is the more general form that accounts for the capacitive current dependance on synaptic conductance time course. It should be noted that at steady state Z_D_ = R_D_. This distance-dependent increase in Z_D_ results progressively larger local EPSPs, which in some morphologies, can combine with an efficient passive propagation of EPSPs to the soma (transfer impedance), thereby counteracting the distance-dependance reduction in the somatic EPSP amplitude due to cable filtering (Jaffe and Carnevale, 1999; Nevian et al., 2007; Schmidt-Hieber et al., 2007). This location independence of EPSP amplitude is also referred to as passive normalization (Jaffe and Carnevale, 1999). Distance-dependent increases in Z_D_ are also thought to be important to increase the probability of evoking a local dendritic spike at distal inputs of basal dendrites of pyramidal neurons, which can then propagate to the soma (Rudolph and Destexhe, 2003).

**Figure 3:**
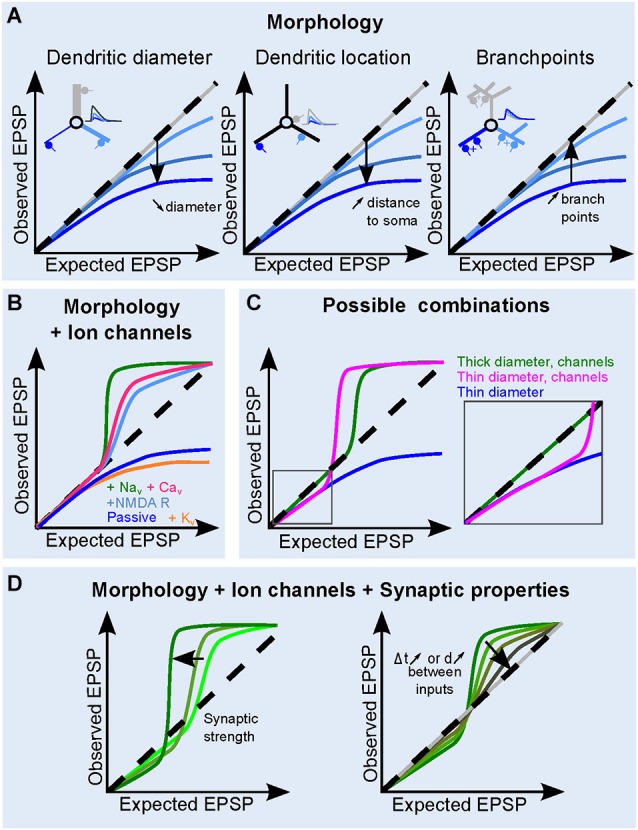
**Contribution of dendritic and synaptic properties to EPSP summation. (A)** Influence of morphological parameters dendritic: diameter (*left*), increasing distance to soma (*middle*) and increasing dendritic branching (*right*) on the dendritic sI/O. The inserts illustrate the effect of morphology on somatic EPSPs under the different conditions. Synapse location and traces are color coded. Dashed line shows a linear I/O for reference. **(B)** The role of ion channels on the shape of the sI/O, for a given morphology. Either K^+^ channels, (*orange*), Na^+^ channels (*green*), VGCC (*pink*), or NMDA receptors (*sky blue*) are added to a passive dendrite (blue). **(C)** Example of sI/O in three realistic combinations: thick (>2 µm) dendrites with active conductances (blue curve, as in Branco and Häusser, 2011), thinner dendrites with active conductances (brown curve, <1 µm, Losonczy and Magee, 2006), or thin dendrites with only passive properties (blue curve, Abrahamsson et al., 2012). **(D)** Influence of synaptic properties on the sI/O for a given morphology and ion channel combination. An increase in synaptic strength makes the sI/O diverge from linearity both in the sublinear and the supralinear regime, whereas increasing the interval or the distance between synaptic inputs tends to linearize the curve (*right*).

Rall provided a simple parameter that describes cable filtering: the space constant (λ), derived from the steady state (λ_DC_) or frequency-dependent (λ_AC_) solution to the cable equations. It represents the distance along a cable where the membrane potential is 63% of the maximal at the site of current injection. Therefore if the dendrite length is longer than λ, significant cable filtering can be expected; similarly, if the dendritic length is much shorter than λ then EPSPs propagating to the soma are filtered very little. A critical morphological parameter determining λ is the dendritic **diameter**, to which λ is proportional (Figure 2B); meaning a larger diameter produces a longer λ (Figure 3A, left). For fast synaptic conductances (rise and decay <2 ms), the capacitive current acts as a frequency-dependent shunt and can dramatically alter λ. In cerebellar molecular layer interneurons, for example, the frequency-dependent length constant (λ_AC_) can be over a factor of 5 shorter than λ_DC_. Their thin (~0.4 µm diameter), 100 µm long dendrites are electrically compact for steady-state depolarizations (with total length 3 times shorter than λ_DC_, 300 µm). But for rapid synaptic conductances λ_AC_ is 50 µm (half the dendritic length), resulting in significant dendritic filtering of EPSPs for distances greater than 20 µm (Abrahamsson et al., 2012). **Dendritic branching** tends to shorten the space constant, since it effectively decreases the membrane resistance (acting like a shunt for current flow (Figure 3A; right; Abrahamsson et al., 2012). It is also worth noting that λ also serves as a rough indicator of the size of effective dendritic compartments. Synapses located within a distance of λ are more likely to interact than non-neighboring synapses (Figure 2C; Abrahamsson et al., 2012).

### The Influence of Passive Dendrites on sI/Os

As described above, sublinear summation of simultaneously occurring EPSPs within an electrical compartment is a natural consequence of the loss of driving force for synaptic currents. Dendritic compartments with narrow diameters are particularly sensitive to this due to a high Z_D_. Therefore when multiple dendritic synapses are activated simultaneously within a close proximity (<λ), the local depolarization resulting from the activation of a given synaptic input will be large, thus decreasing the local driving force, resulting in a sublinear sI/O (Figures 1D, 2B). As the **diameter** of the passive dendrite decreases, Z_D_ will increase and the local EPSPs will be even larger (Abrahamsson et al., 2012). One can use the equation for input resistance of an infinite cable to appreciate the influence of dendritic diameter (Figure 2B, equation 3). The larger Z_D_ causes a larger depolarization, thus the sublinear summation of synaptic inputs will be more prominent with fewer active inputs (Figure 3A, left; see also Rinzel and Rall, 1974). If the **distance of the synapse from the soma** increases, the current sink of the soma, the end effect of the dendrite and/or dendritic tapering will contribute to a distance-dependent increase in Z_D_, together resulting in more pronounced sublinear sI/O curves particularly for more distal dendritic compartments (Figure 3A, middle). Finally, the number of dendritic branch points, despite increasing dendritic filtering, tends to decrease the local Z_D_ by adding a current sink, thus favoring a more linear sI/O (Figure 3A, right). Gap junctions have also been shown to reduce sublinear summation by providing a current sink (Vervaeke et al., 2012).

Although passive membrane properties are sufficient to produce sublinear dendritic operations, experimental evidence of such a mechanism has only recently been described (Abrahamsson et al., 2012; Vervaeke et al., 2012). The authors concluded that the combination of thin dendrites and low levels of expression of voltage-gated channels favors sublinear dendritic operations. In these neurons, sublinear summation is apparent even for as few as two active synapses (Abrahamsson et al., 2012). Synapses activated on separate dendrites summed linearly, supporting a **scatter sensitive** neuronal computation (Abrahamsson et al., 2012), that was confirmed in a realistic active model (Cazé et al., 2013).

### The Influence of Active Dendrites on sI/Os

The large local synaptic depolarizations produced in dendrites can also recruit the activation of voltage-dependent channels (NMDARs, Na^+^, Ca^2+^, K^+^ and HCN channels, see Johnston and Narayanan, 2008; Figure 3B). The number of activated synaptic inputs needed to engage active conductances is determined, in part, by the passive properties of the dendrite, the amplitude and kinetics of the synaptic conductance, the voltage-dependance of channel gating, and the channel density and distribution along the somato-dendritic axis. Active conductances can either enhance (Williams and Stuart, 2000; Migliore and Shepherd, 2002) or dampen (Cash and Yuste, 1999; Hu et al., 2010) local dendritic depolarizations, depending on whether the channels mediate inward (depolarizing) or outward (hyperpolarizing) currents, respectively. Distance-dependent increases in I_h_ currents have been shown to compensate for the temporal slowing caused by dendritic filtering (Magee and Cook, 2000; Williams and Stuart, 2002). Differential expression of HCN channels across mitral cells has also been shown to increase the membrane noise and lower the rheobase, thus facilitating AP generation (Angelo and Margrie, 2011). Because of the presence of NMDARs at many glutamatergic synapses, most studies find that NMDARs activate other voltage-dependent channels by boosting local synaptic depolarization (Schiller et al., 2000; Losonczy and Magee, 2006; Nevian et al., 2007; Makara et al., 2009; Branco and Häusser, 2011; Katona et al., 2011; Krueppel et al., 2011). The resulting dendritic operation is determined by the concurrence of a passively determined sublinear (Losonczy and Magee, 2006; Krueppel et al., 2011; Chiovini et al., 2014) or linear operation (Branco and Häusser, 2011), and a supralinear operation.

In some cases, the voltage activation of conductances results not only in EPSP boosting, but in a threshold-dependent, all-or-none regenerative response, often called a dendritic spike. This regenerative behavior is characterized by a steep change in the sI/O followed by a plateau (Figures 1D, 3B; Polsky et al., 2004; Losonczy and Magee, 2006; Larkum, 2013). Locally-generated dendritic spikes can be mediated by either Na^+^ channels, Ca^2+^ channels or NMDA receptors (NMDARs). Na^+^-spikes are triggered by high-amplitude local depolarization, are relatively brief, and can be accompanied by entry of Ca^2+^ through VGCC or NMDARs. In pyramidal cells, these dendritic Na^+^ spikes can be generated in most regions of the dendritic tree, propagate throughout the dendritic tree, albeit with some attenuation, but can still trigger somatic spiking (Golding and Spruston, 1998; Rudolph and Destexhe, 2003; Nevian et al., 2007). Recent findings have also shown Na^+^-channel dependent spikes in dendrites of dentate gyrus granule cells (Chiovini et al., 2014). On the other hand, Ca^2+^ and NMDA spikes are longer, plateau-like events, that are thought to be generated in particular regions of the dendritic tree, and require the synchronous activation of many clustered synapses. The biophysical mechanisms of the NMDA spikes and their functional consequences have been described in detail in a recent review (Major et al., 2013). In cortical pyramidal neurons, the Ca^2+^ spike is likely to propagate actively from the primary apical dendrite to the soma, thereby representing a more global dendritic operation, whereas NMDA spikes are locally restricted to dendritic compartments such as tufts or basal dendrites (Larkum, 2013). In contrast, simulations of *in vivo* spontaneous synaptic activity allow glutamate-bound NMDARs to act as global nonlinearities providing an entirely different computation than those initiated in single dendrites (Farinella et al., 2014). Nevertheless, several recent *in vivo* studies have reported the involvement of local NMDA spikes during sensory processing, across all layers of the cortex (Lavzin et al., 2012; Xu et al., 2012; Smith et al., 2013; Gambino et al., 2014; Palmer et al., 2014). It should also be noted that Polsky et al. (Polsky et al., 2004) pointed out that a Ca^2+^-spike exhibits saturation of the voltage response and thus can also be considered sublinear for very high stimulation strengths.

In summary, the modus operandi of supralinear dendritic compartments is comprised of a continuum of voltage-dependent operations from simple boosting of synaptic depolarization to regenerative spikes. Considering the biophysical underpinnings of this range of operations, it follows that the interplay of the active and passive properties of dendrites ultimately determines the shape of the sI/O (Figure 3C). For example, sI/Os of thick dendrites, which have a low Z_D_, do not suffer from driving force losses, thus sum linearly for low numbers of activated synapses, then transition into supralinear summation (Makara and Magee, 2013). Thin dendrites on the other hand may exhibit sublinear sI/O relationships for only a few inputs, but then easily engage NMDAR and Ca^2+^ channels (Losonczy and Magee, 2006; Chiovini et al., 2014) with fewer synaptic inputs than in larger dendrites (Figure 3C). Due to tapering of dendritic width, which increases the Z_D_ along the dendrite with increasing distance to the soma, the dendritic operations can be altered as a function of distance from the soma (Branco and Häusser, 2010, 2011).

### The Influence of the Size, Time Course and Location of the Synaptic Conductance on sI/Os

The strength of synaptic conductance varies from synapse to synapse across neuron types, but also within neurons. The **synaptic strength** not only serves to bias the output of a neuron to particular inputs (Ko et al., 2011), but it can also be tuned to compensate for dendritic attenuation by passive dendritic properties (Magee, 2000). Synaptic strength influences dendritic operations by modulating the gain (slope) and shape of the sI/O, which is achieved by engaging sub- and supralinear transformations with different numbers of synaptic inputs (Figure 3D). Larger synaptic conductances will lead to larger dendritic depolarizations, and in turn either a larger reduction in driving force or increased activation of voltage-gated conductances. Depending on the intrinsic membrane properties and synaptic conductance amplitude the “linear regime” may be more or less prominent in the sI/O relationship.

The **temporal window** for synaptic interactions depends ultimately on the time course of local EPSPs, which is itself shaped by the local passive dendritic properties and the time course of the synaptic conductance (Jonas, 2000). Although the local dendritic EPSPs are larger than those at the soma, it is important to note that their time course is generally much faster, due to charge redistribution down the dendrite (Schmidt-Hieber et al., 2007). The degree to which nonlinear mechanisms are engaged during EPSP summation also depends on the temporal summation of local EPSPs (Losonczy and Magee, 2006; Abrahamsson et al., 2012; Makara and Magee, 2013). Simultaneous synaptic activation enables the largest degree of nonlinear summation, which will progressively decrease as the time difference between synaptic events increases (Figures 2A, 3D). Thus, combined with the synaptic strength, the temporal coincidence between co-activated synapses within a single dendritic compartment will determine gain of the dendritic operations (Gómez González et al., 2011; Abrahamsson et al., 2012; Makara and Magee, 2013).

The location of synapses carrying similar information (e.g., a single sensory feature) determines which dendritic mechanism is recruited. For example, if features of an object are always clustered on a single dendritic compartment, then nonlinear summation will be the prominent operation influencing integration. Below we will use a mathematical formalism to provide insight into how **synaptic placement** and dendritic operations influence neuronal computations.

## Experimental Strategies for Studying Dendritic Integration

How do researchers study the biophysical properties of dendrites and their influence on excitatory synaptic integration? Classical electrophysiology methods such as sharp electrode- or patch-clamp-based recordings of somatic membrane potential provided insight into the intrinsic passive electrical properties of neurons by measuring the input resistance and the membrane time constant (τ = R_m_*C_m_) (Spruston and Johnston, 1992). When combined with multi-compartmental dendritic models, with either simplified morphologies (equivalent cylinder approximation) or full anatomical reconstructions (Clements and Redman, 1989; Major et al., 1994), the passive electrotonic properties of dendrites can be estimated from model parameters that predict the membrane potential decay from somatic current injections (Rall et al., 1992). These constrained models are then used to examine dendritic transformations of EPSPs as they propagate to the soma.

Unfortunately, single electrode recordings at the soma do not provide sufficient information about dendritic properties to constrain complex morphological models. With the advent of dendritic patch recordings (Stuart et al., 1993), at least for large diameter dendrites (≥1 µm), cable model predictions could be directly verified. This powerful recording method allows estimations of the critical parameters influencing dendritic filtering, such as internal resistivity (R_i_; Stuart et al., 1993; Stuart and Spruston, 1998; Roth and Häusser, 2001; Nevian et al., 2007; Schmidt-Hieber et al., 2007; Hu et al., 2010), R_m_ and voltage-gated channel properties and density along the somato-dendritic axis (Magee and Johnston, 1995; Stuart and Spruston, 1998; Hu et al., 2010). Dendritic recordings also enabled the measurement of local EPSPs and EPSCs, which allowed the authors to conclude that dendritic filtering can be compensated by a distance-dependent increase in synaptic conductance in certain neuron types (Magee and Cook, 2000).

More recently, fluorescence imaging techniques have greatly increased the toolkit for studying dendritic integration, particularly in those dendrites with narrow diameters (<1 µm). Ca^2+^ indicators are one of the most popular class of fluorescence probes, which are used to indirectly study dendritic nonlinearities resulting from activation of voltage-dependent ion channels, provided at least one type of Ca^2+^ conductance was activated (Markram et al., 1995; Schiller et al., 1995, 1997, 2000). Ca^2+^ indicators have also been used to monitor synaptic activity because of the prevalence of NMDAR activation in single spines and Ca^2+^-permeable AMPARs at synapses in interneurons (Soler-Llavina and Sabatini, 2006). *In vivo* two-photon Ca^2+^ imaging experiments provided the first insights into the spatial and temporal distribution of sensory-evoked synaptic signaling within dendrites (Varga et al., 2011; Lavzin et al., 2012; Smith et al., 2013; Jia et al., 2014; Palmer et al., 2014). The contribution of *in vivo* Ca^2+^ imaging studies to understanding dendritic function has been recently reviewed by Grienberger et al (Grienberger et al., 2015). However, a limitation of using Ca^2+^ imaging to study synaptic integration is that it does not report the true dendritic voltage, a parameter critically influencing dendritic operations. Also, the slow nature of the whole-cell averaged [Ca^2+^] and the use of high affinity Ca^2+^ indicators limits the temporal resolution of this method (Farinella et al., 2014; Fernández-Alfonso et al., 2014). Voltage-sensitive dyes are, in principle, an ideal alternative for direct measurement of dendritic integration. Whereas voltage-sensitive dye recordings have provided unprecedented optical reports of the spatial and temporal distribution of APs in axons (Foust et al., 2010; Popovic et al., 2011) and dendrites (Acker and Antic, 2009; Casale and McCormick, 2011), their use to monitor EPSPs in dendrites has been less successful due to poor signal-to-noise ratio, typically requiring hundreds of trials of averaging (Palmer and Stuart, 2009). However, inhibitory post-synaptic potentials (IPSPs) have been detected (Canepari et al., 2008) and a recent study reports good signal-to-noise ratios sufficient to detect spine EPSPs (Popovic et al., 2014). The advances in genetically-encoded voltage indicators are also rapidly maturing (Hochbaum et al., 2014; St-Pierre et al., 2014; Zou et al., 2014), and could eventually provide a powerful tool for studying dendritic integration *in vivo*.

Another widely-used *in vitro* technique to characterize the integration properties of dendrites is to directly activate postsynaptic receptors using photolysis of caged-neurotransmitter (i.e., caged-glutamate) within the diffraction-limited focal volume of the microscope (Gasparini and Magee, 2006; Losonczy and Magee, 2006). Using galvanometer-driven mirrors, the type regularly used in scanning confocal microscopy, the focal illumination volume can be rapidly moved (within 0.1–1 ms) and positioned at multiple locations. The uncaging light pulse is then rapidly gated at each location to focally release glutamate. This allows for the near simultaneous activation of many postsynaptic sites. The somatic depolarization is then recorded using standard whole-cell patch-clamp methods. The observed response to uncaging at multiple synaptic locations (typically within 1 ms) is compared to the arithmetic sum of the uncaging-evoked responses at individual sites. The resulting plot is identical to the sI/O plots described in Figures 1, 3, provided that the uncaging responses are similar to synaptic activation. Using light, rather than presynaptic vesicular release, to activate neurotransmitter receptors provides a more flexible strategy to systematically vary the number, pattern, and timing of synapse activation. Electrical stimulation does not permit a precise identification of the synapses being activated, nor precise control of the number of synapses activated. Holographic illumination provides an alternative strategy for true simultaneous glutamate uncaging at multiple sites within the dendrites and is more amenable to multibranch activation (Lutz et al., 2008; Yang et al., 2014, 2011). The only potential drawback of uncaging is the difficulty in some preparations to accurately reproduce very fast synaptic conductances due to the large volume of diffraction-limited focal spots relative to the point source nature of neurotransmitter release from synaptic vesicles (DiGregorio et al., 2007), as well as a tendency to partially block GABARs (Fino et al., 2009). Nevertheless, neurotransmitter uncaging is an essential tool for quantifying the biophysical properties underlying dendritic operations.

## Linking Dendritic Operations to Neuronal Computations Using Mathematical Models

Because experimental evidence of a direct link between the dendritic operations and the associated neuronal computations is still lacking, a parallel strategy is to use analytical models to make testable predictions (Poirazi and Mel, 2001; Legenstein and Maass, 2011; Cazé et al., 2013). These methods take advantage of mathematical approximations of measured dendritic operations to make estimates of the possible number and type of neuronal computations. Biophysical models, in contrast, although explicit, do not easily provide insight into the classes of possible computations because of the large parameter space. There is no doubt that such models have provided deep insights into neuronal computations that involve nonlinear dendritic operations. They have been used to show that neurons with supralinear dendrites are** cluster-sensitive** (Mel, 1993) and neurons with sublinear dendrites are **scatter-sensitive** (Koch et al., 1983; Cazé et al., 2013). Yet it was not clear whether either type of nonlinearity provides similar computational advantages. To examine the difference between supralinear and sublinear operations of binary neuron models Cazé et al. (2013) used a Boolean-based analysis. Here we review how this Boolean framework can be used to argue that either supralinear or sublinear summation is sufficient to endow neurons with a new class of computations.

**Figure 4:**
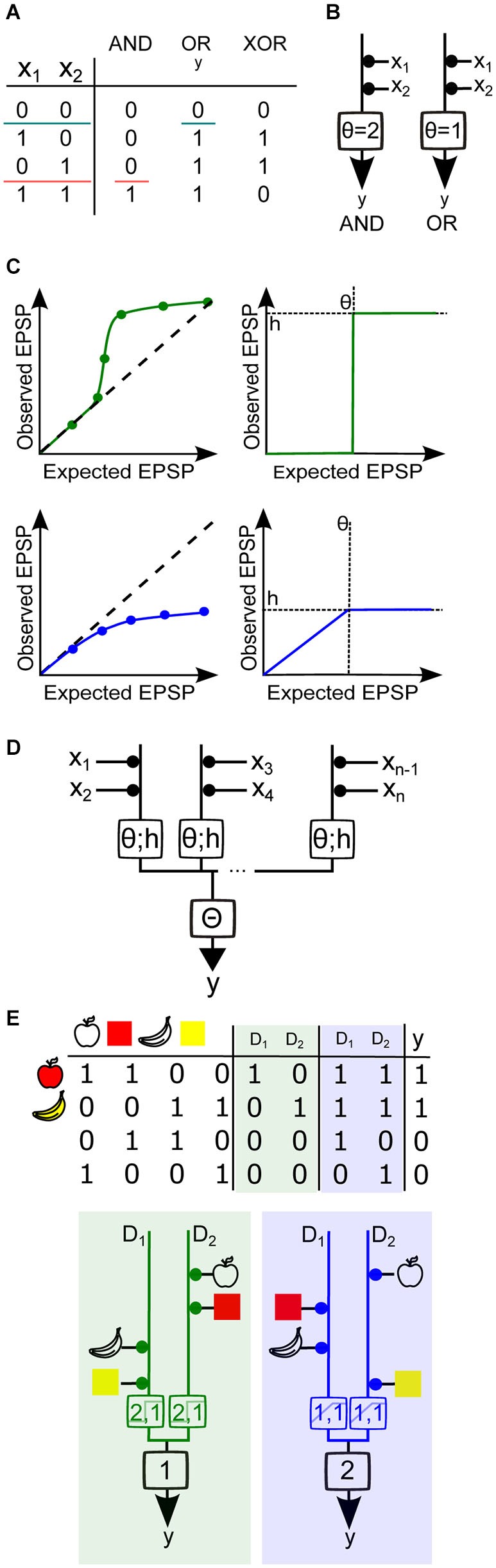
**Using Boolean algebra to analyze binary neuron models with dendritic nonlinearities. (A)** Truth tables for the Boolean functions AND, OR and XOR for two synaptic inputs (*x*_1_ and *x*_2_). The two colored horizontal lines illustrate how the AND and OR functions are linearly separable, i.e., a single line divides all inputs between two groups, one group having an output of 0 and the other group having an output of 1. Neuron output binary value is denoted as *y*. **(B)** Threshold linear unit model neuron with two inputs. The weight of each input is represented by the area of the black disc drawn between the input and the model neuron. Here all weights are equal to 1. A spiking threshold (θ) of 2 allows the model neuron to compute the AND function (*left*), whereas if θ =1 the neuron computes the OR function (*right*). **(C)** *Top*, Simplified representations of a supralinear sI/O (*left*) and its mathematical approximation by a Heaviside function (*right*) with a height h and a threshold θ. *Bottom*, simplified representation of a sublinear sI/O and its mathematical approximation by a piecewise linear, then saturating function. **(D)** Generalized diagram representing a two-layer integration model neuron with several compartments and n inputs. Each branch represents a dendritic compartment, and the integration operation performed by this compartment is represented by the box on the branch. The threshold θ and the output value h of the nonlinearity are indicated within the box. The result from the integration from each branch is then linearly summed and compared to the somatic spike threshold Θ. **(E)** Implementation of the (partial) feature binding problem (pFBP) by binary neurons with two dendritic compartments D_1_ and D_2_, either supralinear or sublinear. *Top*, truth table describing various input feature combinations, the response of each dendritic compartment, D (0:inactive/1:active), and the final neuronal output, *y*. Columns with green shading are the outputs of dendrites exhibiting supralinear operations, while columns shaded in blue contain outputs of dendrites that exhibit sublinear operations. *Bottom*, Model neuron with equivalent dendrite representation that can implement the pFBP using supralinear (*left*) or sublinear dendritic compartments (*right*), with θ and h values indicated in the box. If dendritic integration is supralinear, two groups of inputs are needed to activate a compartment, and a single compartment can trigger a spike. If dendritic integration is sublinear, a single input can activate the dendritic compartment and the two compartments must be active to trigger a spike.

Within this analytical framework, neurons are modeled as having binary inputs (x_i_), which can be weighted and integrated, resulting in binary outputs (y). In this context the input-output relation is described by a unique truth table, corresponding to a Boolean function. In Figure 4A, the truth table describes three simple Boolean functions: OR, AND and XOR. This well-known mathematical framework (Wegener, 1987; Crama and Hammer, 2011), which deals with binary classifications of binary words, allows us to analytically determine what type of classifications are possible with nonlinear dendrites and which are otherwise impossible.

The simplest binary neuron model is called the threshold linear unit, also known as the point neuron model as described first by McCulloch and Pitts (Figure 4B; McCulloch and Pitts, 1943). Synapses are assigned a binary value of 0 or 1, for inactive or active states, which is then multiplied by a positive synaptic weight for excitatory synapses. The sum of the active weighted inputs is then compared to a somatic spike threshold Θ. If this weighted sum is greater than the threshold, the output is assigned a value of 1, and otherwise zero. If one considers a neuron with linearly summing excitatory inputs, adjustment of the threshold allows it to either perform a Boolean AND or OR (Figure 4B). However, it is not possible to find a threshold value and positive synaptic weight that allows the computation of the XOR, the function corresponding to a binary neuron that would fire only when one synapse is active, but not otherwise. This illustrates well the fact that the threshold linear unit can only perform functions that are linearly separable, i.e., there is a set of weights and a spike threshold that categorizes the inputs into two distinct groups, which differ by their output values (Figure 4A). The XOR does not meet this criterion and is therefore a part of the class of functions that are linearly non-separable. To solve this problem we must either invoke a non-monotone function to combine synaptic values (Zador et al., 1992) or consider synaptic inhibition by using negative weights (Mel, 1994; Cazé et al., 2014). Because the former has not been described experimentally, and the latter requires specific wiring within the network, we will focus here on linearly non-separable functions that can be implemented with only excitatory synapses and monotone dendritic operations. These functions are known as positive Boolean functions (Cazé et al., 2013).

Linearly–separable functions represent only a small fraction of all the possible computations (Cazé et al., 2013). However, a neuron with nonlinear dendritic compartments can implement the set of linearly non-separable functions, which encompasses a much larger fraction of all computations (Cazé et al., 2013). Thus both supralinear and sublinear compartments unlock the access to all the possible computations (Mel and Koch, 1990; Mel, 1991). This formal result is true for an infinite number of dendritic compartments (Poirazi and Mel, 2001). This is clearly impossible in practice. So what can a neuron compute with a finite number of dendritic compartments?

To address this question we can construct a two-layer binary model with nonlinear dendritic compartments. We first approximated the dendritic sI/O with functions each having a characteristic dendritic threshold **θ**, which represents the threshold of the dendritic nonlinearity, and **h**, which represents the maximal value of the dendritic nonlinearity. To approximate supralinear compartments we used a Heaviside function, and for sublinear functions we used a piecewise linear saturating function (Figure 4C). The output of the dendritic compartments is then linearly summed and compared with spike threshold (Figure 4D). If we vary synaptic weights, the thresholds, and the nonlinear dendritic operations, we can use Boolean analysis to examine the different functions this model can implement. A functionally salient neuronal computation that requires dendritic nonlinearities is the association (or binding) of two features of an object (for example, their shape and color). This is known as the feature binding problem (FBP). If we suppose that that different features of objects are encoded by different groups of pre synaptic neurons impinging on the same post synaptic neuron, then it is obvious that by allowing the features of an object to target the same supralinear dendrite, the coincidence of those features can be easily detected when co-active (i.e., “red” + “apple shape”; Figure 4E). It can also be shown that a sublinear operation can bind features if the inputs that encode object features are distributed onto different dendritic subunits (and the spiking threshold increased). From these simple binary models it is again clear that supralinear operations favor cluster sensitivity and sublinear operations favor scatter sensitivity. However, a keen eye may notice that the sublinear model will also produce a spike if the apple shape and banana shape are both activated. This therefore constitutes a partial FBP. Below we will describe a neuron model with equivalent dendrites that can implement the complete FBP.

**Figure 5:**
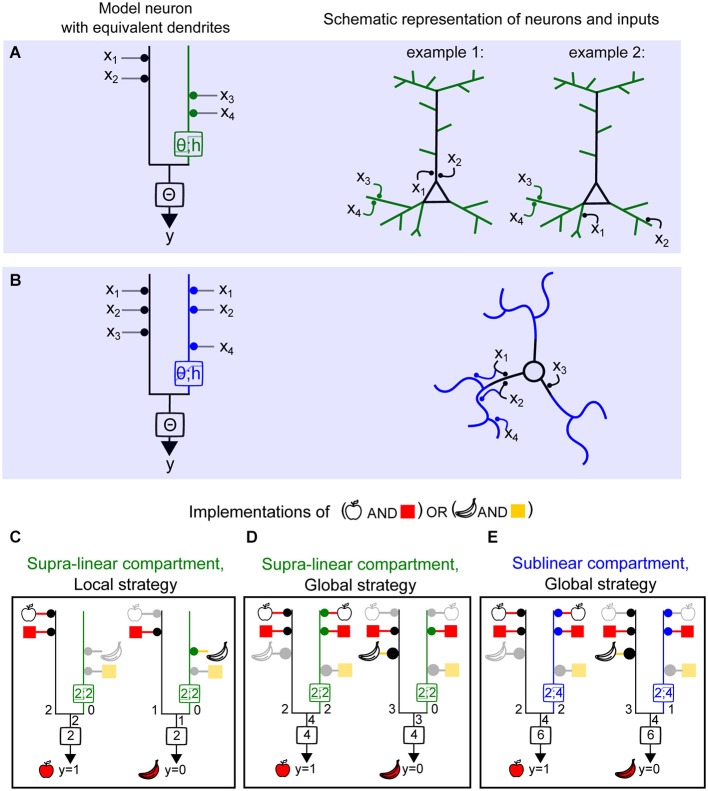
**Computing a linearly non-separable function (full FBP) with supralinear and sublinear dendrites and using local vs. global synaptic wiring strategies**. **(A)** *Left*, model neuron with equivalent dendrite representation of two compartments, linear (black) and supralinear (green), and a clustered distribution of object features (object 1 : ×1, ×2 and object 2 : ×3, x4) (local strategy). *Right*, schematic representations of synaptic placements equivalent to the model on the left. **(B)** *Left*, model neuron with equivalent dendrite representation of two compartments, linear (black) and sublinear (blue), and a distributed placement of inputs carrying object features. **(C–E)** Implementation of the full FBP (*y* = 1; “apple shape and red” or “banana shape and yellow”). **(C)**, implementation of the full FBP using a model with a supralinear compartment and a local wiring strategy. Inactive inputs are represented in light gray and the corresponding feature in lighter color. **(D)** Implementation of the full FBP using a model with a supralinear compartment and a global wiring strategy. The area of the disc adjacent to a compartment next to each object feature represents the relative weight of this feature. Here the relative weights used are of 1 and 2. **(E)** Implementation of the full FBP using a model with sublinear compartment and a global wiring strategy.

Because neurons are known to have both linear and nonlinear compartments, we considered how more realistic dendritic trees could be represented using our simple binary model, by creating a neuron model with equivalent dendrites (Figures 5A,B). All linear regions of the dendritic tree (typically, the perisomatic compartment or the large diameter primary dendrites) were collapsed to a single equivalent “linear” compartment (black regions of schematic neuron and left branch of the model neuron). The nonlinear dendritic compartments receiving more than one synaptic input were represented as a second equivalent dendritic branch. This then generalizes to an equivalent dendritic branch for each nonlinear electrical compartment (Figure 4D). The presence of a linear compartment is important, since inputs contacting two separate nonlinear dendrites will sum linearly (Figure 5A). Also, even inputs contacting the same nonlinear dendrite, provided they are not in the same electrical compartment, will sum linearly.

Legenstein et al. demonstrated that a model neuron with supralinear dendritic integration is capable of learning and computing the FBP (Legenstein and Maass, 2011). This function detects any correct combination of features for an object, but not incorrect combinations. In the Boolean framework this would be the truth table corresponding to (“red” + “apple shape”) or (“yellow” + “banana shape”). In Figure 4 we showed that two supralinear dendrites are sufficient to solve the FBP for two objects made of two features each. In Figure 5, a neuron displaying at least one supralinear compartment and a linear compartment can also solve the FBP for four inputs. In this case, inputs encoding the features of one object are assigned to the supralinear compartment, and the features corresponding to the other object are assigned to the linear compartment (Figure 5C). Because the features of the object must “cluster” on the same compartment we refer to this model as using a **local strategy** of computation. Interestingly, it is also possible to implement the same computation using a **global strategy**, meaning that the features corresponding to one object need to be “scattered” onto both the nonlinear and the linear compartment (Figure 5D), provided that appropriate changes in the synaptic weights and threshold values are also implemented. As shown by Cazé et al. (2013), a model with a linear and sublinear compartment requires the global strategy to perform the FBP (Figure 5E). The synaptic weights and threshold will also be different than in the case of a model neuron with a supralinear compartment. The fact that the FBP can be implemented using a global strategy contrasts with the notion that recognition of an object required the clustering of the inputs carrying its features onto a same dendritic branch (Legenstein and Maass, 2011), and the assumption that two-layer integration models require independent branch-specific operations (Behabadi and Mel, 2014). Using a biophysical model with a model stellate cell morphology, Cazé et al. showed the predictions are robust, since only passive thin dendrites were necessary to convey a scatter sensitivity of output firing, even in the presence of synaptic noise (Cazé et al., 2013).

How might simplified Boolean models be modified for more features and/or more objects? For objects represented by more than two features, clustered strategies would simply require more synaptic inputs, such that the number of the number of inputs per subunit (dendritic compartment) equals the number features. A change in threshold would also be required. The requirements for neuronal computations using sublinear dendrites, however, depend on the type of computation and are less straightforward to determine explicitly. The necessary number of nonlinear subunits also varies given the implementation strategy, the number of objects, the type of nonlinear subunits and the number of features. To solve the FBP with more objects using supralinear operations, each object will require at least one subunit (Cazé et al., 2012). For computations with sublinear operations, Cazé et al. showed that using binary weights, the FBP requires a maximum of 2*^n^* subunits (Cazé et al., 2012). Considering non-binary weights then reduces the number of subunits needed, but this number is still higher than the number of necessary supralinear subunits (*n*_subunits_ = *n*_objects_).

In summary, neurons with sublinear dendrites are capable of solving linearly non-separable functions, but require using a distributed strategy of synaptic placement (Figure 5E). These neurons will be scatter sensitive. On the other hand, neurons with supralinear dendrites can also access the same class of computations either by using this strategy (Figure 5C) or by clustering functionally relevant inputs onto the same compartment (Figure 5C). Hence they can be either scatter or cluster sensitive. Thus, the final neuronal computations depend not only on the type of dendritic operation and the dendritic and axosomatic thresholds, but also the *global* mapping of input features throughout the dendritic tree.

## Open Questions

To understand how a neuron integrates its synaptic inputs we need precise knowledge of the morphology, ion channel distribution along the tree, strength and time course of synaptic conductances carrying particular information features, the output spike threshold, and the spatio-temporal pattern of activation of the synapses carrying these features. Although we can determine most of these parameters, as we reviewed above, the most challenging experiments are those designed to estimate the spatio-temporal distribution of all synapses carrying relevant sensory features (i.e., a functional connectivity map). Strategies using injection of viral-based retrograde tracers (Marshel et al., 2010) are powerful for the identification of connected presynaptic cells, but these methods lack information about features conveyed by the inputs. Using *in vivo* Ca^2+^ imaging, researchers have begun the herculean task of estimating how sensory features are mapped onto dendritic trees by examining how single synapses and dendrites respond to behavioral stimuli. It is not clear whether such feature mapping can be performed on the entire dendritic tree, but initial results provide hints as to whether there may be general mapping rules. Some studies argue that features are clustered in single dendrites within the somato-sensory cortex (Takahashi et al., 2012), consistent with a **local computation strategy**, while other studies have shown that neighboring synapses onto layer 2/3 pyramidal neurons of the visual and auditory cortex respond maximally for activation of inputs carrying different sensory features (Jia et al., 2010; Chen et al., 2011), consistent with a **global strategy**. In light of the conclusions described here, both computation strategies are capable of performing linearly non-separable functions.

Why might neurons use different dendritic operations and wiring strategies? It is conceivable that differences in timing of sensory development or optimal local circuit wiring may constrain wiring strategies for particular neurons. Thus to perform the same computation, different wiring and dendritic strategies are needed. Global wiring strategies are more amenable to “random wiring,” in contrast to the specific connectivity required for engaging local strategies. We speculate that different dendritic operations may be implemented by neurons given certain biological constraints, such as limitations in the number and location of synapses carrying a particular feature, or spike threshold. For example when both principal neurons and interneurons receive a common set of input features along relatively fixed axonal projections, but are required to perform different computations, they may engage different dendritic operations. In the cerebellum interneurons have been shown to exhibit sublinear dendritic operations (Abrahamsson et al., 2012; Vervaeke et al., 2012) on their parallel fiber inputs, while Purkinje cells are thought to receive the same or similar features from the same set of input fibers, yet display supralinear dendritic operations (Rancz and Häusser, 2006). One could speculate that the different nonlinearities and synaptic placement strategies of Purkinje neurons and interneurons may enable them to implement complementary computations, which ultimately could result in a microcircuit that is highly selective for specific input patterns.

What are the wiring rules? Three possible wiring strategies are (1) predetermined connectivity (genetically encoded); (2) random connectivity; and (3) activity-dependent pruning and stabilization of connections. Although the exact contribution of each mechanism is yet to be determined, synaptic plasticity has been shown to modify and ultimately determine the functional connectivity. For example computational modeling showed that a local wiring strategy, in which synapses carrying features of objects are clustered, can be learned using simple plasticity rules (Legenstein and Maass, 2011). Experimental evidence supports this theoretical work, suggesting that activity-dependent, branch-specific plasticity strengthens clustered synaptic inputs and their compartmentalization (Makara et al., 2009; Makino and Malinow, 2011; Takahashi et al., 2012). On the other hand, synaptic plasticity could also reinforce global computational strategies. In cerebellar stellate cells, high-frequency firing of clustered inputs has been described to induce profound presynaptic short- and long-term synaptic depression (Beierlein and Regehr, 2006; Soler-Llavina and Sabatini, 2006). Such plasticity mechanisms would reinforce the neuron’s **scatter sensitivity**, and thus tend to optimize the output firing for specific spatially and temporally sparse synaptic activity patterns (Abrahamsson et al., 2012; Cazé et al., 2013).

Synchronized neuronal activity is known to cause oscillations of the dendritic voltage, which would inevitably reinforce electrical interactions between dendrites and thus alter the effective number of isolated dendritic subunits that contribute to the neuronal computation. For example, Remme et al. (2009) showed theoretically that input-dependent synchronization of intrinsic dendritic voltage oscillations can facilitate global voltage propagation, even throughout highly distributed dendritic trees. It will be important to examine how local and global dendritic integration strategies might be influenced by brain oscillations, thus ultimately altering neuronal and even circuit computations.

Since many types of interneurons are known to contact specific locations within the dendritic tree, inhibition will undoubtedly influence integration properties and information processing by neuronal circuits (as reviewed by Palmer et al., 2012). Nevertheless, the experimental challenge is to determine not only the timing and location of inhibition within the dendrite, in order to determine their alteration of dendritic operations, but also whether particular features are conveyed similarly or differently by excitatory and inhibitory inputs. Although complex, the problem is critical to understanding brain function as the balance of excitation and inhibition is well known to be tightly regulated, with alterations being implicated in disease (Yizhar et al., 2011). Using the Boolean analysis of equivalent dendrites, one can deduce that negative weight associated with inhibition is capable of performing the Boolean NOT function. Such a function would enable a simple implementation of XOR computations, further expanding the number of computable linearly non-separable functions.

## Summary

In this review we described categories of biophysical and cellular mechanisms that influence dendritic operations: passive and active membrane properties of the dendritic tree, the time course and amplitude of synaptic activation, the output spike threshold, and finally the location and pattern of the activation of synaptic inputs. We discussed how each of these parameters shapes and tunes the sI/O. We briefly discussed techniques for the characterization of dendritic operations, including electrode-based methods to stimulate and/or record from dendrites, optical techniques to image dendritic activity or uncage neurotransmitter, and biophysical modeling. In order to understand how the major classes of dendritic operations (linear, sublinear and supralinear) link to neuronal computations, we reviewed the use of binary models associated with Boolean analysis. This analysis provides insight into the types of computable neuronal functions, such as the object feature binding problem. We also reviewed how such functions can be implemented with either supralinear or sublinear dendrites depending on the spatial mapping of those features within the dendritic tree. Because the synaptic activity pattern ultimately determines the neuronal computations, we propose that the elemental computational unit is the neuron rather than the dendrite (Cazé et al., 2014). Although there are cases (local strategies) where dendritic operations can dictate the neuronal computation, dendritic operations must be studied and understood in the context of the knowledge of the wiring of specific features onto the dendritic tree.

## Conflict of Interest Statement

The authors declare that the research was conducted in the absence of any commercial or financial relationships that could be construed as a potential conflict of interest.

## References

[B1] Abrahamsson T. Cathala L. Matsui K. Shigemoto R. Digregorio D. A. (2012). Thin dendrites of cerebellar interneurons confer sublinear synaptic integration and a gradient of short-term plasticity. Neuron 73, 1159–1172. 10.1016/j.neuron.2012.01.027 22445343

[B2] Acker C. D. Antic S. D. (2009). Quantitative assessment of the distributions of membrane conductances involved in action potential backpropagation along basal dendrites. J. Neurophysiol. 101, 1524–1541. 10.1152/jn.00651.2007 19118105 PMC2666409

[B3] Agmon-Snir H. Carr C. Rinzel J. (1998). The role of dendrites in auditory coincidence detection. Nature 393, 268–272. 10.1038/30505 9607764

[B4] Angelo K. Margrie T. W. (2011). Population diversity and function of hyperpolarization-activated current in olfactory bulb mitral cells. Sci. Rep. 1:50. 10.1038/srep00050 22355569 PMC3216537

[B5] Archie K. A. Mel B. W. (2000). A model for intradendritic computation of binocular disparity. Nat. Neurosci. 3, 54–63. 10.1038/71125 10607395

[B6] Behabadi B. F. Mel B. W. (2014). Mechanisms underlying subunit independence in pyramidal neuron dendrites. Proc. Natl. Acad. Sci. U S A 111, 498–503. 10.1073/pnas.1217645111 24357611 PMC3890819

[B7] Beierlein M. Regehr W. G. (2006). Local interneurons regulate synaptic strength by retrograde release of endocannabinoids. J. Neurosci. 26, 9935–9943. 10.1523/jneurosci.0958-06.2006 17005857 PMC6674464

[B8] Branco T. Häusser M. (2010). The single dendritic branch as a fundamental functional unit in the nervous system. Curr. Opin. Neurobiol. 20, 494–502. 10.1016/j.conb.2010.07.009 20800473

[B9] Branco T. Häusser M. (2011). Synaptic integration gradients in single cortical pyramidal cell dendrites. Neuron 69, 885–892. 10.1016/j.neuron.2011.02.006 21382549 PMC6420135

[B10] Canepari M. Vogt K. Zecevic D. (2008). Combining voltage and calcium imaging from neuronal dendrites. Cell. Mol. Neurobiol. 28, 1079–1093. 10.1007/s10571-008-9285-y 18500551 PMC3143714

[B11] Casale A. E. McCormick D. A. (2011). Active action potential propagation but not initiation in thalamic interneuron dendrites. J. Neurosci. 31, 18289–18302. 10.1523/JNEUROSCI.4417-11.2011 22171033 PMC3269759

[B12] Cash S. Yuste R. (1998). Input summation by cultured pyramidal neurons is linear and position-independent. J. Neurosci. 18, 10–15. 9412481 10.1523/JNEUROSCI.18-01-00010.1998 PMC6793421

[B13] Cash S. Yuste R. (1999). Linear summation of excitatory inputs by CA1 pyramidal neurons. Neuron 22, 383–394. 10.1016/s0896-6273(00)81098-3 10069343

[B14] Cazé R. D. Humphries M. D. Gutkin B. S. (2012). Spiking and saturating dendrites differentially expand single neuron computation capacity. Adv. Neural Inf. Process. Syst. 25, 1–9.

[B15] Cazé R. D. Humphries M. Gutkin B. (2013). Passive dendrites enable single neurons to compute linearly non-separable functions. PLoS Comput. Biol. 9:e1002867. 10.1371/journal.pcbi.1002867 23468600 PMC3585427

[B16] Cazé R. D. Humphries M. D. Gutkin B. S. (2014). “Dendrites enhance both single neuron and network computation,” in The Computing Dendrite: From Structure to Function, eds Cuntz H. Remme M. W. H. Torben-Nielsen B. (New York: Springer), 365–380.

[B17] Chadderton P. Schaefer A. T. Williams S. R. Margrie T. W. (2014). Sensory-evoked synaptic integration in cerebellar and cerebral cortical neurons. Nat. Rev. Neurosci. 15, 71–83. 10.1038/nrn3648 24434910

[B18] Chen X. Leischner U. Rochefort N. L. Nelken I. Konnerth A. (2011). Functional mapping of single spines in cortical neurons in vivo. Nature 475, 501–505. 10.1038/nature10193 21706031

[B19] Chiovini B. Turi G. F. Katona G. Kaszás A. Pálfi D. Maák P.. (2014). Dendritic spikes induce ripples in parvalbumin interneurons during hippocampal sharp waves. Neuron 82, 908–924. 10.1016/j.neuron.2014.04.004 24853946

[B20] Clements J. D. Redman S. J. (1989). Cable properties of cat spinal motoneurones measured by combining voltage clamp, current clamp and intracellular staining. J. Physiol. 409, 63–87. 10.1113/jphysiol.1989.sp017485 2585300 PMC1190432

[B21] Crama Y. Hammer P. L. (2011). Boolean Functions. Theory, Algorithms and Applications. New York: Cambridge University Press.

[B22] DiGregorio D. A. Rothman J. S. Nielsen T. A. Silver R. A. (2007). Desensitization properties of AMPA receptors at the cerebellar mossy fiber granule cell synapse. J. Neurosci. 27, 8344–8357. 10.1523/jneurosci.2399-07.2007 17670981 PMC6147216

[B23] Farinella M. Ruedt D. T. Gleeson P. Lanore F. Silver R. A. (2014). Glutamate-bound NMDARs arising from in vivo-like network activity extend spatio-temporal integration in a L5 cortical pyramidal cell model. PLoS Comput. Biol. 10:e1003590. 10.1371/journal.pcbi.1003590 24763087 PMC3998913

[B24] Fernández-Alfonso T. Nadella K. M. Iacaruso M. F. Pichler B. Roš H. Kirkby P. A.. (2014). Monitoring synaptic and neuronal activity in 3D with synthetic and genetic indicators using a compact acousto-optic lens two-photon microscope. J. Neurosci. Methods 222, 69–81. 10.1016/j.jneumeth.2013.10.021 24200507 PMC3889102

[B25] Fino E. Araya R. Peterka D. S. Saliemo S. Etchenique R. Yuste R. (2009). RuBi-Glutamate: two-photon and visible-light photoactivation of neurons and dendritic spines. Front. Neural Circuits 3:2. 10.3389/neuro.04.002.2009 19506708 PMC2691658

[B26] Foust A. Popovic M. Zecevic D. McCormick D. A. (2010). Action potentials initiate in the axon initial segment and propagate through axon collaterals reliably in cerebellar Purkinje neurons. J. Neurosci. 30, 6891–6902. 10.1523/JNEUROSCI.0552-10.2010 20484631 PMC2990270

[B27] Gambino F. Pagès S. Kehayas V. Baptista D. Tatti R. Carleton A.. (2014). Sensory-evoked LTP driven by dendritic plateau potentials in vivo. Nature 515, 116–119. 10.1038/nature13664 25174710

[B28] Gasparini S. Magee J. C. (2006). State-dependent dendritic computation in hippocampal CA1 pyramidal neurons. J. Neurosci. 26, 2088–2100. 10.1523/jneurosci.4428-05.2006 16481442 PMC6674927

[B29] Golding N. L. Spruston N. (1998). Dendritic sodium spikes are variable triggers of axonal action potentials in hippocampal CA1 pyramidal neurons. Neuron 21, 1189–1200. 10.1016/s0896-6273(00)80635-2 9856473

[B30] Gómez González J. Mel B. W. Poirazi P. (2011). Distinguishing linear vs. Non-linear integration in CA1 radial oblique dendrites: it’s about time. Front. Comput. Neurosci. 5:44. 10.3389/fncom.2011.00044 22171217 PMC3214726

[B31] Grienberger C. Chen X. Konnerth A. (2015). Dendritic function in vivo. Trends Neurosci. 38, 45–54. 10.1016/j.tins.2014.11.002 25432423

[B32] Häusser M. Mel B. (2003). Dendrites: bug or feature? Curr. Opin. Neurobiol. 13, 372–383. 10.1016/s0959-4388(03)00075-8 12850223

[B33] Hochbaum D. R. Zhao Y. Farhi S. L. Klapoetke N. Werley C. A. Kapoor V.. (2014). All-optical electrophysiology in mammalian neurons using engineered microbial rhodopsins. Nat. Methods 11, 825–833. 10.1038/nmeth.3000 24952910 PMC4117813

[B34] Hu H. Martina M. Jonas P. (2010). Dendritic mechanisms underlying rapid synaptic activation of fast-spiking hippocampal interneurons. Science 327, 52–58. 10.1126/science.1177876 19965717

[B35] Jaffe D. B. Carnevale N. T. (1999). Passive normalization of synaptic integration influenced by dendritic architecture. J. Neurophysiol. 82, 3268–3285. 10601459 10.1152/jn.1999.82.6.3268

[B36] Jia H. Rochefort N. L. Chen X. Konnerth A. (2010). Dendritic organization of sensory input to cortical neurons in vivo. Nature 464, 1307–1312. 10.1038/nature08947 20428163

[B37] Jia H. Varga Z. Sakmann B. Konnerth A. (2014). Linear integration of spine Ca2+ signals in layer 4 cortical neurons in vivo. Proc. Natl. Acad. Sci. U S A 111, 9277–9282. 10.1073/pnas.1408525111 24927564 PMC4078833

[B38] Johnston D. Narayanan R. (2008). Active dendrites: colorful wings of the mysterious butterflies. Trends Neurosci. 31, 309–316. 10.1016/j.tins.2008.03.004 18471907

[B39] Jonas P. (2000). The time course of signaling at central glutamatergic synapses. News Physiol. Sci. 15, 83–89. 11390884 10.1152/physiologyonline.2000.15.2.83

[B40] Katona G. Kaszás A. Turi G. F. Hájos N. Tamás G. Vizi E. S.. (2011). Roller Coaster Scanning reveals spontaneous triggering of dendritic spikes in CA1 interneurons. Proc. Natl. Acad. Sci. U S A 108, 2148–2153. 10.1073/pnas.1009270108 21224413 PMC3033272

[B41] Ko H. Hofer S. B. Pichler B. Buchanan K. A. Sjöström P. J. Mrsic-Flogel T. D. (2011). Functional specificity of local synaptic connections in neocortical networks. Nature 473, 87–91. 10.1038/nature09880 21478872 PMC3089591

[B42] Koch C. Poggio T. Torre V. (1983). Nonlinear interactions in a dendritic tree: localization, timing and role in information processing. Proc. Natl. Acad. Sci. U S A 80, 2799–2802. 10.1073/pnas.80.9.2799 6573680 PMC393916

[B43] Krueppel R. Remy S. Beck H. (2011). Dendritic integration in hippocampal dentate granule cells. Neuron 71, 512–528. 10.1016/j.neuron.2011.05.043 21835347

[B44] Larkum M. (2013). A cellular mechanism for cortical associations: an organizing principle for the cerebral cortex. Trends Neurosci. 36, 141–151. 10.1016/j.tins.2012.11.006 23273272

[B45] Lavzin M. Rapoport S. Polsky A. Garion L. Schiller J. (2012). Nonlinear dendritic processing determines angular tuning of barrel cortex neurons in vivo. Nature 490, 397–401. 10.1038/nature11451 22940864

[B46] Lee D. Lin B. J. Lee A. K. (2012). Hippocampal place fields emerge upon single-cell manipulation of excitability during behavior. Science 337, 849–853. 10.1126/science.1221489 22904011

[B47] Legenstein R. Maass W. (2011). Branch-specific plasticity enables self-organization of nonlinear computation in single neurons. J. Neurosci. 31, 10787–10802. 10.1523/JNEUROSCI.5684-10.2011 21795531 PMC6623094

[B48] London M. Häusser M. (2005). Dendritic computation. Annu. Rev. Neurosci. 28, 503–532. 10.1146/annurev.neuro.28.061604.135703 16033324

[B49] Longordo F. To M.-S. Ikeda K. Stuart G. J. (2013). Sublinear integration underlies binocular processing in primary visual cortex. Nat. Neurosci. 16, 714–723. 10.1038/nn.3394 23644484

[B50] Losonczy A. Magee J. C. (2006). Integrative properties of radial oblique dendrites in hippocampal CA1 pyramidal neurons. Neuron 50, 291–307. 10.1016/j.neuron.2006.03.016 16630839

[B51] Lutz C. Otis T. S. DeSars V. Charpak S. DiGregorio D. A. Emiliani V. (2008). Holographic photolysis of caged neurotransmitters. Nat. Methods 5, 821–827. 10.1038/nmeth.1241 19160517 PMC2711023

[B52] Magee J. C. (2000). Dendritic integration of excitatory synaptic input. Nat. Rev. Neurosci. 1, 181–190. 10.1038/35044552 11257906

[B53] Magee J. C. Cook E. P. (2000). Somatic EPSP amplitude is independent of synapse location in hippocampal pyramidal neurons. Nat. Neurosci. 3, 895–903. 10.1038/78800 10966620

[B54] Magee J. C. Johnston D. (1995). Characterization of single voltage-gated Na+ and Ca2+ channels in apical dendrites of rat CA1 pyramidal neurons. J. Physiol. 487(Pt. 1), 67–90. 10.1113/jphysiol.1995.sp020862 7473260 PMC1156600

[B55] Major G. Larkman A. U. Jonas P. Sakmann B. Jack J. J. (1994). Detailed passive cable models of whole-cell recorded CA3 pyramidal neurons in rat hippocampal slices. J. Neurosci. 14, 4613–4638. 8046439 10.1523/JNEUROSCI.14-08-04613.1994 PMC6577163

[B56] Major G. Larkum M. E. Schiller J. (2013). Active properties of neocortical pyramidal neuron dendrites. Annu. Rev. Neurosci. 36, 1–24. 10.1146/annurev-neuro-062111-150343 23841837

[B57] Makara J. K. Losonczy A. Wen Q. Magee J. C. (2009). Experience-dependent compartmentalized dendritic plasticity in rat hippocampal CA1 pyramidal neurons. Nat. Neurosci. 12, 1485–1487. 10.1038/nn.2428 19898470

[B58] Makara J. K. Magee J. C. (2013). Variable dendritic integration in hippocampal CA3 pyramidal neurons. Neuron 80, 1438–1450. 10.1016/j.neuron.2013.10.033 24360546 PMC3878388

[B59] Makino H. Malinow R. (2011). Compartmentalized versus global synaptic plasticity on dendrites controlled by experience. Neuron 72, 1001–1011. 10.1016/j.neuron.2011.09.036 22196335 PMC3310180

[B60] Markram H. Helm P. J. Sakmann B. (1995). Dendritic calcium transients evoked by single back-propagating action potentials in rat neocortical pyramidal neurons. J. Physiol. 485(Pt. 1), 1–20. 10.1113/jphysiol.1995.sp020708 7658365 PMC1157968

[B61] Marshel J. H. Mori T. Nielsen K. J. Callaway E. M. (2010). Targeting single neuronal networks for gene expression and cell labeling in vivo. Neuron 67, 562–574. 10.1016/j.neuron.2010.08.001 20797534 PMC2929426

[B62] Martin A. R. (1955). A further study of the statistical composition on the end-plate potential. J. Physiol. 130, 114–122. 10.1113/jphysiol.1955.sp005397 13278890 PMC1363457

[B63] McCulloch W. S. Pitts W. (1943). A logical calculus of the ideas immanent in nervous activity. Bull. Math. Biol. 5, 115–133 10.1007/bf02478259 2185863

[B64] Mel B. W. (1991). A connectionist model may shed light on neural mechanisms for visually guided reaching. J. Cogn. Neurosci. 3, 273–292. 10.1162/jocn.1991.3.3.273 23964842

[B65] Mel B. W. (1992). NMDA-based pattern discrimination in a modeled cortical neuron. Neural Comput. 4, 502–517 10.1162/neco.1992.4.4.502

[B66] Mel B. W. (1993). Synaptic integration in an excitable dendritic tree. J. Neurophysiol. 70, 1086–1101. 8229160 10.1152/jn.1993.70.3.1086

[B67] Mel B. W. (1994). Information processing in dendritic trees. Neural Comput. 6, 1031–1085 10.1162/neco.1994.6.6.1031

[B68] Mel B. W. Koch C. (1990). “Sigma-pi learning: on radial basis functions and cortical associative learning,” in Advances in Neural Information Processing Systems, ed Touretzky D. S. (San Mateo, CA: Morgan Kaufmann), 474–481.

[B69] Mel B. W. Ruderman D. L. Archie K. A. (1998). Translation-invariant orientation tuning in visual “complex” cells could derive from intradendritic computations. J. Neurosci. 18, 4325–4334. 9592109 10.1523/JNEUROSCI.18-11-04325.1998 PMC6792789

[B70] Migliore M. Shepherd G. M. (2002). Emerging rules for the distributions of active dendritic conductances. Nat. Rev. Neurosci. 3, 362–370. 10.1038/nrn810 11988775

[B71] Nevian T. Larkum M. E. Polsky A. Schiller J. (2007). Properties of basal dendrites of layer 5 pyramidal neurons: a direct patch-clamp recording study. Nat. Neurosci. 10, 206–214. 10.1038/nn1826 17206140

[B72] Palmer L. Murayama M. Larkum M. (2012). Inhibitory regulation of dendritic activity in vivo. Front. Neural Circuits 6:26. 10.3389/fncir.2012.00026 22654734 PMC3360463

[B74] Palmer L. M. Shai A. S. Reeve J. E. Anderson H. L. Paulsen O. Larkum M. E. (2014). NMDA spikes enhance action potential generation during sensory input. Nat. Neurosci. 17, 383–390. 10.1038/nn.3646 24487231

[B73] Palmer L. M. Stuart G. J. (2009). Membrane potential changes in dendritic spines during action potentials and synaptic input. J. Neurosci. 29, 6897–6903. 10.1523/JNEUROSCI.5847-08.2009 19474316 PMC6665597

[B75] Poirazi P. Mel B. W. (2001). Impact of active dendrites and structural plasticity on the memory capacity of neural tissue. Neuron 29, 779–796. 10.1016/s0896-6273(01)00252-5 11301036

[B76] Polsky A. Mel B. W. Schiller J. (2004). Computational subunits in thin dendrites of pyramidal cells. Nat. Neurosci. 7, 621–627. 10.1038/nn1253 15156147

[B77] Popovic M. A. Foust A. J. McCormick D. A. Zecevic D. (2011). The spatio-temporal characteristics of action potential initiation in layer 5 pyramidal neurons: a voltage imaging study. J. Physiol. 589, 4167–4187. 10.1113/jphysiol.2011.209015 21669974 PMC3180577

[B78] Popovic M. A. Gao X. Carnevale N. T. Zecevic D. (2014). Cortical dendritic spine heads are not electrically isolated by the spine neck from membrane potential signals in parent dendrites. Cereb. Cortex 24, 385–395. 10.1093/cercor/bhs320 23054810 PMC3888368

[B79] Rall W. (1967). Distinguishing theoretical synaptic potentials computed for different soma-dendritic distributions of synaptic input. J. Neurophysiol. 30, 1138–1168. 6055351 10.1152/jn.1967.30.5.1138

[B80] Rall W. Burke R. E. Holmes W. R. Jack J. J. Redman S. J. Segev I. (1992). Matching dendritic neuron models to experimental data. Physiol. Rev. 72, S159–S186. 1438585 10.1152/physrev.1992.72.suppl_4.S159

[B81] Rancz E. A. Häusser M. (2006). Dendritic calcium spikes are tunable triggers of cannabinoid release and short-term synaptic plasticity in cerebellar Purkinje neurons. J. Neurosci. 26, 5428–5437. 10.1523/jneurosci.5284-05.2006 16707795 PMC5886360

[B82] Remme M. W. H. Lengyel M. Gutkin B. S. (2009). The role of ongoing dendritic oscillations in single-neuron dynamics. PLoS Comput. Biol. 5:e1000493. 10.1371/journal.pcbi.1000493 19730677 PMC2725317

[B83] Rinzel J. Rall W. (1974). Transient response in a dendritic neuron model for current injected at one branch. Biophys. J. 14, 759–790. 10.1016/s0006-3495(74)85948-5 4424185 PMC1334571

[B84] Roth A. Häusser M. (2001). Compartmental models of rat cerebellar Purkinje cells based on simultaneous somatic and dendritic patch-clamp recordings. J. Physiol. 535, 445–472. 10.1111/j.1469-7793.2001.00445.x 11533136 PMC2278793

[B85] Rudolph M. Destexhe A. (2003). A fast-conducting, stochastic integrative mode for neocortical neurons in vivo. J. Neurosci. 23, 2466–2476. 12657707 10.1523/JNEUROSCI.23-06-02466.2003 PMC6742032

[B86] Schiller J. Helmchen F. Sakmann B. (1995). Spatial profile of dendritic calcium transients evoked by action potentials in rat neocortical pyramidal neurones. J. Physiol. 487(Pt. 3), 583–600. 10.1113/jphysiol.1995.sp020902 8544123 PMC1156647

[B87] Schiller J. Major G. Koester H. J. Schiller Y. (2000). NMDA spikes in basal dendrites of cortical pyramidal neurons. Nature 404, 285–289. 10.1038/35005094 10749211

[B88] Schiller J. Schiller Y. Stuart G. Sakmann B. (1997). Calcium action potentials restricted to distal apical dendrites of rat neocortical pyramidal neurons. J. Physiol. 505(Pt. 3), 605–616. 10.1111/j.1469-7793.1997.605ba.x 9457639 PMC1160039

[B89] Schmidt-Hieber C. Jonas P. Bischofberger J. (2007). Subthreshold dendritic signal processing and coincidence detection in dentate gyrus granule cells. J. Neurosci. 27, 8430–8441. 10.1523/jneurosci.1787-07.2007 17670990 PMC6673070

[B90] Silver R. A. (2010). Neuronal arithmetic. Nat. Rev. Neurosci. 11, 474–489. 10.1038/nrn2864 20531421 PMC4750293

[B91] Smith S. L. Smith I. T. Branco T. Häusser M. (2013). Dendritic spikes enhance stimulus selectivity in cortical neurons in vivo. Nature 503, 115–120. 10.1038/nature12600 24162850 PMC6319606

[B92] Soler-Llavina G. J. Sabatini B. L. (2006). Synapse-specific plasticity and compartmentalized signaling in cerebellar stellate cells. Nat. Neurosci. 9, 798–806. 10.1038/nn1698 16680164

[B93] Spruston N. (2008). Pyramidal neurons: dendritic structure and synaptic integration. Nat. Rev. Neurosci. 9, 206–221. 10.1038/nrn2286 18270515

[B94] Spruston N. Johnston D. (1992). Perforated patch-clamp analysis of the passive membrane properties of three classes of hippocampal neurons. J. Neurophysiol. 67, 508–529. 1578242 10.1152/jn.1992.67.3.508

[B95] Spruston N. Kath W. L. (2004). Dendritic arithmetic. Nat. Neurosci. 7, 567–569. 10.1038/nn0604-567 15162161

[B96] St-Pierre F. Marshall J. D. Yang Y. Gong Y. Schnitzer M. J. Lin M. Z. (2014). High-fidelity optical reporting of neuronal electrical activity with an ultrafast fluorescent voltage sensor. Nat. Neurosci. 17, 884–889. 10.1038/nn.3709 24755780 PMC4494739

[B97] Stuart G. J. Dodt H. U. Sakmann B. (1993). Patch-clamp recordings from the soma and dendrites of neurons in brain slices using infrared video microscopy. Pflugers Arch. 423, 511–518. 10.1007/bf00374949 8351200

[B98] Stuart G. Spruston N. (1998). Determinants of voltage attenuation in neocortical pyramidal neuron dendrites. J. Neurosci. 18, 3501–3510. 9570781 10.1523/JNEUROSCI.18-10-03501.1998 PMC6793161

[B99] Takahashi N. Kitamura K. Matsuo N. Mayford M. Kano M. Matsuki N.. (2012). Locally synchronized synaptic inputs. Science 335, 353–356. 10.1126/science.1210362 22267814

[B100] Varga Z. Jia H. Sakmann B. Konnerth A. (2011). Dendritic coding of multiple sensory inputs in single cortical neurons in vivo. Proc. Natl. Acad. Sci. U S A 108, 15420–15425. 10.1073/pnas.1112355108 21876170 PMC3174623

[B101] Vervaeke K. Lorincz A. Nusser Z. Silver R. A. (2012). Gap junctions compensate for sublinear dendritic integration in an inhibitory network. Science 335, 1624–1628. 10.1126/science.1215101 22403180 PMC3587282

[B102] Wegener I. (1987). The Complexity of Boolean Functions. New York, NY: John Wiley & Sons, Inc.

[B103] Williams S. R. Stuart G. J. (2000). Site independence of EPSP time course is mediated by dendritic I(h) in neocortical pyramidal neurons. J. Neurophysiol. 83, 3177–3182. 10805715 10.1152/jn.2000.83.5.3177

[B104] Williams S. R. Stuart G. J. (2002). Dependence of EPSP efficacy on synapse location in neocortical pyramidal neurons. Science 295, 1907–1910. 10.1126/science.1067903 11884759

[B105] Xu N.-L. Harnett M. T. Williams S. R. Huber D. O’Connor D. H. Svoboda K.. (2012). Nonlinear dendritic integration of sensory and motor input during an active sensing task. Nature 492, 247–251. 10.1038/nature11601 23143335

[B106] Yang S. Emiliani V. Tang C. M. (2014). The kinetics of multibranch integration on the dendritic arbor of CA1 pyramidal neurons. Front. Cell Neurosci. 8:127. 10.3389/fncel.2014.00127 24860429 PMC4026731

[B107] Yang S. Papagiakoumou E. Guillon M. de Sars V. Tang C. M. Emiliani V. (2011). Three-dimensional holographic photostimulation of the dendritic arbor. J. Neural Eng. 8:046002. 10.1088/1741-2560/8/4/046002 21623008

[B108] Yizhar O. Fenno L. E. Prigge M. Schneider F. Davidson T. J. O’Shea D. J.. (2011). Neocortical excitation/inhibition balance in information processing and social dysfunction. Nature 477, 171–178. 10.1038/nature10360 21796121 PMC4155501

[B109] Zador A. M. Claiborne B. J. Brown T. J. (1992). “Nonlinear pattern separation in single hippocampal neurons with active dendritic membrane,” in Advances in Neural Information Processing Systems, eds Moody J. Hanson S. Lippman R. (Cambridge, MA: Morgan Kaufmann).

[B110] Zou P. Zhao Y. Douglass A. D. Hochbaum D. R. Brinks D. Werley C. A.. (2014). Bright and fast multicoloured voltage reporters via electrochromic FRET. Nat. Commun. 5:4625. 10.1038/ncomms5625 25118186 PMC4134104

